# Research on the characteristics of electro-hydraulic position servo system of RBF neural network under fuzzy rules

**DOI:** 10.1038/s41598-024-64262-7

**Published:** 2024-07-03

**Authors:** Jianying Li, Weidong Li, Xiaoyan Du

**Affiliations:** 1 School of Mechanical and Power Engineering, Harbin University of Science and Technology, Harbin, 150080 China; 2 Key Laboratory of Advanced Manufacturing and Intelligent Technology, Ministry of Education, Harbin, 150080 China

**Keywords:** Electro-hydraulic position servo system, RBF neural network, Fuzzy rules, PID parameter learning rate, Phase lag, Load stiffness, Electrical and electronic engineering, Mechanical engineering

## Abstract

A radial basis function neural network PID controller under fuzzy rules (FUZZY-RBF-PID) was designed for the electro-hydraulic position servo system under the influence of uncertain factors such as load mutation, and load stiffness change. Firstly, the mathematical model of the system is established, and the frequency domain and time domain analysis of the system are carried out. Secondly, based on the analysis results, a radial basis function (RBF) neural network PID controller is designed, and fuzzy rules are innovatively used to adjust the learning rate of PID parameters in the RBF neural network learning algorithm in real time. Thirdly, the simulation results show that under the action of the FUZZY-RBF-PID controller, the unit step response of the system has high steady-state accuracy, fast response speed, and under the condition of large load stiffness, the system can recover to the steady-state value faster after being disturbed. At the same time, when the input signal is the sinusoidal signal of 10 HZ, the system under the action of the FUZZY-RBF-PID controller has no obvious phase lag phenomenon, and the tracking error is minimal. The proposed method can effectively improve the comprehensive performance of the electro-hydraulic position servo system under the influence of uncertain factors.

## Introduction

Electro-hydraulic servo system (EHSS) has the advantages of high power density, fast response speed, and high control accuracy, which is widely used in aerospace, weapons and equipment, civil industry, and other fields, such as pitch system^1^, mechanical arm^2^, excavator^3^ and so on. However, several factors affect the control accuracy of EHSS. In industrial production, it is difficult to determine the speed and acceleration signals, and there is noise in the detection signal, and the controller is difficult to achieve the established effect. Wang et al^4^. proposed an output feedback controller based on the condition that only displacement signals are available. It can reduce the influence of measurement noise and complete the precise position control of EHSS. However, the estimation speed signal by the Levant differentiator is seriously affected by noise. Using an extended state observer (ESO) to estimate the speed signal is more efficient, and the disturbance can be effectively estimated at the same time^5^. EHSS is subject to external load disturbance during operation, and it is often difficult to ensure control accuracy under disturbance conditions. Designing a disturbance observer or ESO can provide a good estimation of the disturbance^6^. Sliding mode control can effectively suppress the disturbance^7^, but the convergence time of the sliding mode surface cannot be guaranteed^8^. The terminal sliding mode controller can eliminate the above drawbacks and enhance the convergence performance^9^. In addition, parameter uncertainty often exists in EHSS, which also seriously affects the control accuracy of the system. Parameter adaptive control, adaptive robust control, and dynamic surface control proposed to avoid the “differential explosion” problem of backstepping can deal with parameter unknown and perturbation problems^10–12^. However, the design of the above control strategies and disturbance observation methods depends on the accuracy of the system model and the need to acquire apriori knowledge of the system parameters and boundaries^13,14^. In engineering applications, it is often difficult to obtain the above information, when the work requirements and conditions change, some system parameters will change, and the above modern control methods can not play an advantage. Due to the advantages of not relying on accurate mathematical models and easy operation, PID controllers are widely used in engineering applications. However, with the development of science and technology, the control requirements for EHSS are also increasing, and the traditional PID controller can not meet the growing needs, so the combination of intelligent control can make the control effect further, the particle swarm optimization algorithm and other optimization algorithms combined with PID controller^15^, in the system with little change in working conditions have a good play. However, when the system is subject to external disturbance and the system parameters are uncertain, the PID controller parameters should be adjusted online to obtain better results.

Radial basis function (RBF) neural networks have the characteristics of strong real-time, strong self-learning ability, and strong adaptive ability. Combining it with a PID controller to obtain an RBF neural network PID controller can solve the problem that the traditional algorithm is not robust. RBF-PID controller adjusts the RBF neural network parameters according to the deviation between the system output value and the identified output value, identifies the Jacobian information of the controlled object online, and then completes the adjustment of PID parameters. RBF-PID controller has a wide range of applications and certain beneficial effects. Zhang et al.^16^ and Li et al.^17^ applied it to a hot blast furnace and wind turbine blade mold heating system, which can well track the changing temperature control parameters. Dong and Wei^18^ applied it to the automatic train driving system, divided the train running speed according to different running sections, and used the RBF-PID controller to control the train running speed. The output speed was taken as the input parameter of the RBF-PID controller, and the related information (exhaust pressure and exhaust temperature) of the speed was added as the input parameters of the controller to control the speed of the diesel generator^19^. Xiong et al.^20^ and Liu et al.^21^ took the angle deviation value as the control target to control the grader and the quadrotor aircraft respectively. Liu et al.^22^ applied RBF-PID controller to pneumatic artificial muscle and EHSS respectively to control the position of pneumatic tendon and hydraulic cylinder, which can obtain high control accuracy^23^. However, the RBF-PID controller has the problem that it is difficult to select the parameters of the neural network and PID learning rate. Improper selection of parameters will lead to poor control effect and excessive overshoot. Reference^24^ is a traditional RBF-PID controller, which does not consider the problem of learning rate. Fei and Wu^25^ used the improved beetle antennae search algorithm to optimize the parameters of the neural network, completed PID parameter adjustment on this basis, and achieved certain beneficial effects. Reference^26^ used the particle swarm algorithm to optimize the neural network parameters in a fuzzy RBF neural network PID controller. The above two methods can find the optimal neural network parameters under the current conditions, for the adjustment rate of the PID parameters is fixed. However, when the system parameters are perturbed or subjected to uncertain external disturbance, the adjustment rate of the PID parameters can not be intelligently corrected according to the amplitude of the disturbance suffered and the parameter perturbation, which will cause the problem of slow adjustment or overshooting oscillation.

Fuzzy rules can solve such problems as adjustment conflict and parameter selection. The input parameters are interpreted by a membership function, and the output vectors are assigned according to simple fuzzy rules^27^. The gain in the sliding mode controller is used to compensate for the vibration and uncertainty caused by the controller. Too much value of gain will lead to frequent switching and affect the control effect. Fuzzy rules were used to select this value according to the deviation^28^. Fuzzy rules were applied to the cooperative control system of multiple independent robots to arbitrate and balance the conflicts between robots^29^. The selection of the scaling factor of the input and output parameters in the FUZZY-PID controller is very important. Fuzzy rules were used to adjust the selection of the scaling factors according to the deviation and the deviation change rate^30^.

Neural networks have strong self-learning ability, but poor reasoning ability. Fuzzy rules cannot study by themselves, but they have strong logical reasoning abilities. Therefore, fuzzy rules can be combined with neural networks^31,32^. In this paper, fuzzy rules will be used to adjust the learning rate of PID parameters in the RBF-PID control algorithm online. The learning rate is intelligently adjusted according to the degree of disturbance and uncertainty, which improves the learning speed of the neural network, suppresses the phenomenon of overshooting oscillation, and further improves the comprehensive performance of the RBF-PID controller.

## Model of electro-hydraulic position servo system

**Figure 1:**
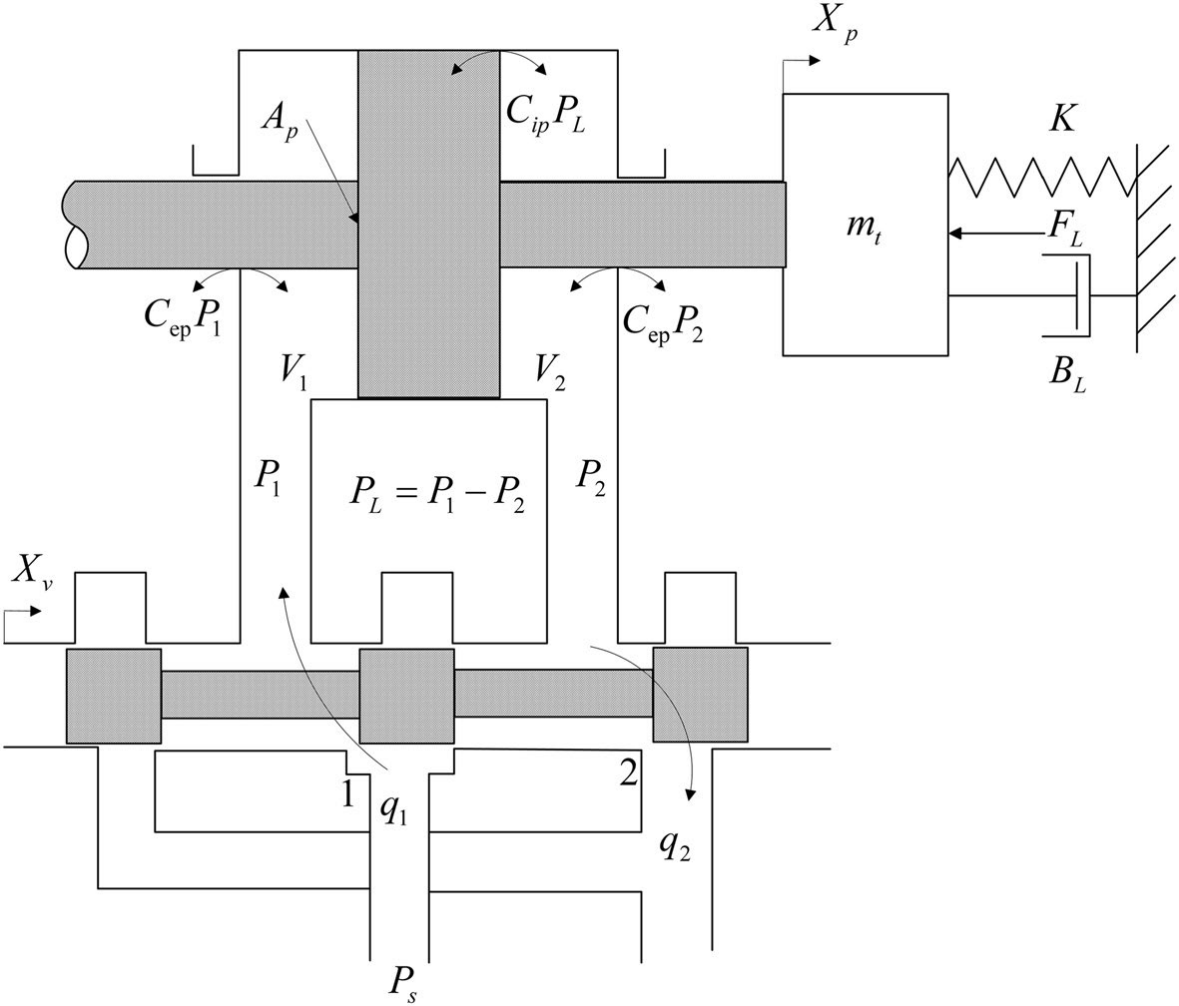
Four-way valve control hydraulic cylinder schematic diagram.

**Figure 2:**
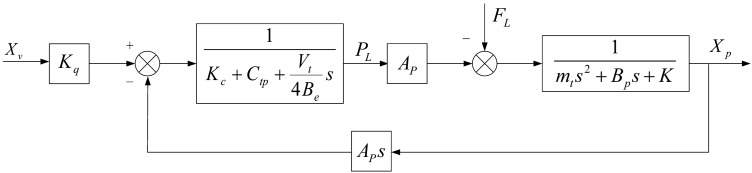
Block diagram of displacement output of valve-controlled hydraulic cylinder.

The schematic diagram of the four-way valve control hydraulic cylinder is shown in Fig. 1.

1 $$ Q_{L} = K_{q} X_{v} - K_{c} P_{L} $$

2 $$ Q_{L} = A_{q} sX_{p} - C_{tp} P_{L} + \frac{{V_{t} }}{{4\beta_{e} }}P_{L} $$

3 $$ A_{p} P_{L} = m_{t} s^{2} X_{p} + B_{p} sX_{p} + KX_{p} + F_{L} $$

where $F_{L}$ is load(N). According to Eqs. (1), (2) and (3), there is block diagram of displacement output of valve-controlled hydraulic cylinder which is shown as Fig. 2.

4 $$ X_{p} = \frac{{\frac{{K_{q} }}{{A_{p} }}X_{v} - \frac{{K_{ce} }}{{A_{p}^{2} }}\left( {1 + \frac{{V_{t} }}{{4\beta_{e} K_{ce} }}s} \right)F_{L} }}{{\frac{{m_{t} V_{t} }}{{4\beta_{e} A_{p}^{2} }}s^{3} + \left( {\frac{{m_{t} K_{ce} }}{{A_{p}^{2} }} + \frac{{B_{p} V_{t} }}{{4\beta_{e} A_{p}^{2} }}} \right)s^{2} + \left( {1 + \frac{{B_{p} K_{ce} }}{{A_{p}^{2} }} + \frac{{KV_{t} }}{{4\beta_{e} A_{p}^{2} }}} \right)s + \frac{{KK_{ce} }}{{A_{p}^{2} }}}} $$

where $K_{ce}$ is the total flow-pressure coefficient, $K_{ce} = K_{c} + C_{tp}$. According to Eq. (4), there is:

5 $$ \frac{Y}{U} = \frac{{\frac{{4\beta_{e} K_{q} A_{p} }}{{m_{t} V_{t} }}}}{{s^{3} + \left( {\frac{{4\beta_{e} K_{ce} }}{{V_{t} }} + \frac{{B_{p} }}{{m_{t} }}} \right)s^{2} + \left( {\frac{{4\beta_{e} A_{p}^{2} }}{{m_{t} V_{t} }} + \frac{{4\beta_{e} B_{p} K_{ce} }}{{m_{t} V_{t} }} + \frac{K}{{m_{t} }}} \right)s + \frac{{4\beta_{e} KK_{ce} }}{{m_{t} V_{t} }}}} $$

The system nominal parameters of the electro-hydraulic position servo system used for simulation research are shown in Table 1.

6 $$ \frac{Y}{U} = \frac{{1.55556 \times 10^{8} }}{{s^{3} + 1.50297277 \times 10^{3} \times s^{2} + 9.48667 \times 10^{6} \times s + 4954618}} $$

**Table 1:** System nominal parameter table.

Notation	Specification	Value
$A_{q}$	Effective area of the piston rod of the hydraulic cylinder (m^2^)	0.1256
$B_{p}$	Viscous damping coefficient of piston and load (N s/m)	2.25 × 10^6^
$m_{t}$	Total mass of piston and load converted to piston (kg)	1500
$C_{tp}$	The total leakage coefficient of the hydraulic cylinder (m^5^/(N m))	5 × 10^−16^
$K$	Load spring stiffness (N/m)	2.5 × 10^9^
$K_{q}$	Flow gain (m^2^/s)	2.5
$K_{c}$	Flow pressure coefficient (m^5^/(N·m))	4 × 10^−12^
$\beta_{e}$	Effective elastic bulk modulus (Pa)	7 × 10^8^
$V_{t}$	Total volume of two cavities (m^3^)	3.768 × 10^−3^

Take the state variables as:

7 $$ X_{1} = X_{p} ,X_{2} = \dot{X}_{p} ,X_{3} = P_{L} $$

The input variable is $$\user2{u = }\left[ {\begin{array}{*{20}c} 0 & {{\varvec{F}}_{{\varvec{L}}} } & {{\varvec{X}}_{{\varvec{v}}} } \\ \end{array} } \right]^{T}$$, from Eqs. (1)–(3):

8 $$ \dot{X}_{1} = X_{2} $$

9 $$ \dot{X}_{2} = \frac{{A_{p} }}{{m_{t} }}X_{3} - \frac{{B_{p} }}{{m_{t} }}X_{2} - \frac{K}{{m_{t} }}X_{1} - \frac{{F_{L} }}{{m_{t} }} $$

10 $$ \dot{X}_{3} = \frac{{4\beta_{e} K_{q} }}{{V_{t} }}X_{v} - \frac{{4\beta_{e} }}{{V_{t} }}(C_{tp} + K_{c} )X_{3} - \frac{{4\beta_{e} A_{p} }}{{V_{t} }}X_{2} $$

Represent it as an equation of state space:

11 $$ \left[ {\begin{array}{*{20}c} {\dot{X}_{1} } \\ {\dot{X}_{2} } \\ {\dot{X}_{3} } \\ \end{array} } \right] = \dot{X} = AX + Bu = \left[ {\begin{array}{*{20}c} 0 & 1 & 0 \\ { - \frac{K}{{m_{t} }}} & { - \frac{{B_{p} }}{{m_{t} }}} & {\frac{{A_{p} }}{{m_{t} }}} \\ 0 & { - \frac{{4\beta_{e} A_{p} }}{{V_{t} }}} & { - \frac{{4\beta_{e} }}{{V_{t} }}(C_{tp} + K_{c} )} \\ \end{array} } \right]\left[ {\begin{array}{*{20}c} {X_{1} } \\ {X_{2} } \\ {X_{3} } \\ \end{array} } \right] + \left[ {\begin{array}{*{20}c} 0 & 0 & 0 \\ 0 & { - \frac{1}{{m_{t} }}} & 0 \\ 0 & 0 & {\frac{{4\beta_{e} K_{q} }}{{V_{t} }}} \\ \end{array} } \right]\left[ {\begin{array}{*{20}c} 0 \\ {F_{L} } \\ {X_{v} } \\ \end{array} } \right] $$

12 $$ {\varvec{Y}} = {\varvec{CX}} = \left[ {\begin{array}{*{20}c} 1 & 0 & 0 \\ 0 & 1 & 0 \\ 0 & 0 & 1 \\ \end{array} } \right]\left[ {\begin{array}{*{20}c} {{\varvec{X}}_{1} } \\ {{\varvec{X}}_{2} } \\ {{\varvec{X}}_{3} } \\ \end{array} } \right] = \left[ {\begin{array}{*{20}c} {{\varvec{X}}_{1} } \\ {{\varvec{X}}_{2} } \\ {{\varvec{X}}_{3} } \\ \end{array} } \right] $$

## Characteristic analysis of electro-hydraulic position servo system

**Figure 3:**
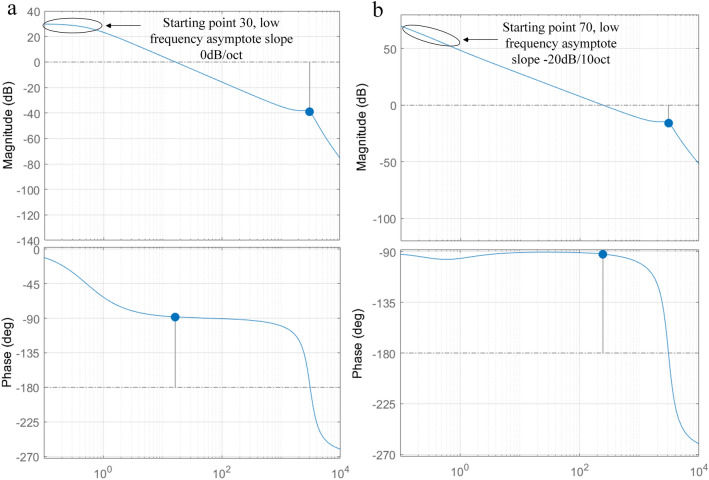
Open loop Bode plot: (**a**) System open-loop Bode diagram; (**b**) PID control of open loop Bode diagram.

Draw the open-loop Bode diagram of the system without controller as shown in Fig. 3. The crossing Angle frequency of the system is 16.4 rad/s and the phase margin is 91.7°, At a frequency of 3080 rad/s, the gain margin is 39.2 dB. The system is stable, the zeros and poles are distributed to the left of the imaginary axis or at the origin, which is a minimum phase Angle system. The low-frequency starting point of the bode diagram of the system’s open loop is 30, and the asymptote slope is 0 dB/Oct. The steady state accuracy of the system is poor.

Apply a PID controller to the system, and the open-loop Bode diagram of the system under PID control was drawn. The crossing frequency of the system was 247 rad/s, and the phase margin was 87.7°. The gain margin is 15.7 dB at a frequency of 3080 rad/s. The starting point of the low frequency band of the Bode chart is 70, and the asymptote slope is − 20 dB/Oct. The steady-state accuracy of the system is improved to a certain extent.

**Figure 4:**
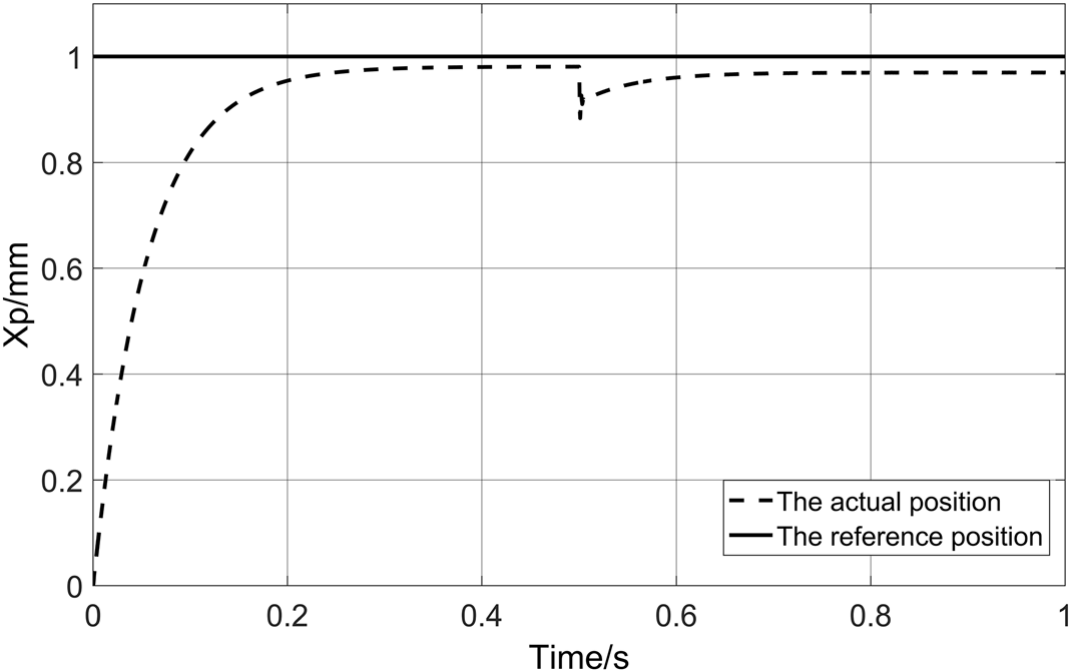
Response curve of load state.

Given the step signal of the system, external load disturbance is applied to the system at 0.5 s, and the response curve of the system is shown in Fig. 4. It can be seen that the time for the system to reach the stable state is 0.45 s, and there is a certain steady-state error, the fluctuation amplitude of the disturbance is 9.8%, and the adjustment time is 0.33 s.

**Figure 5:**
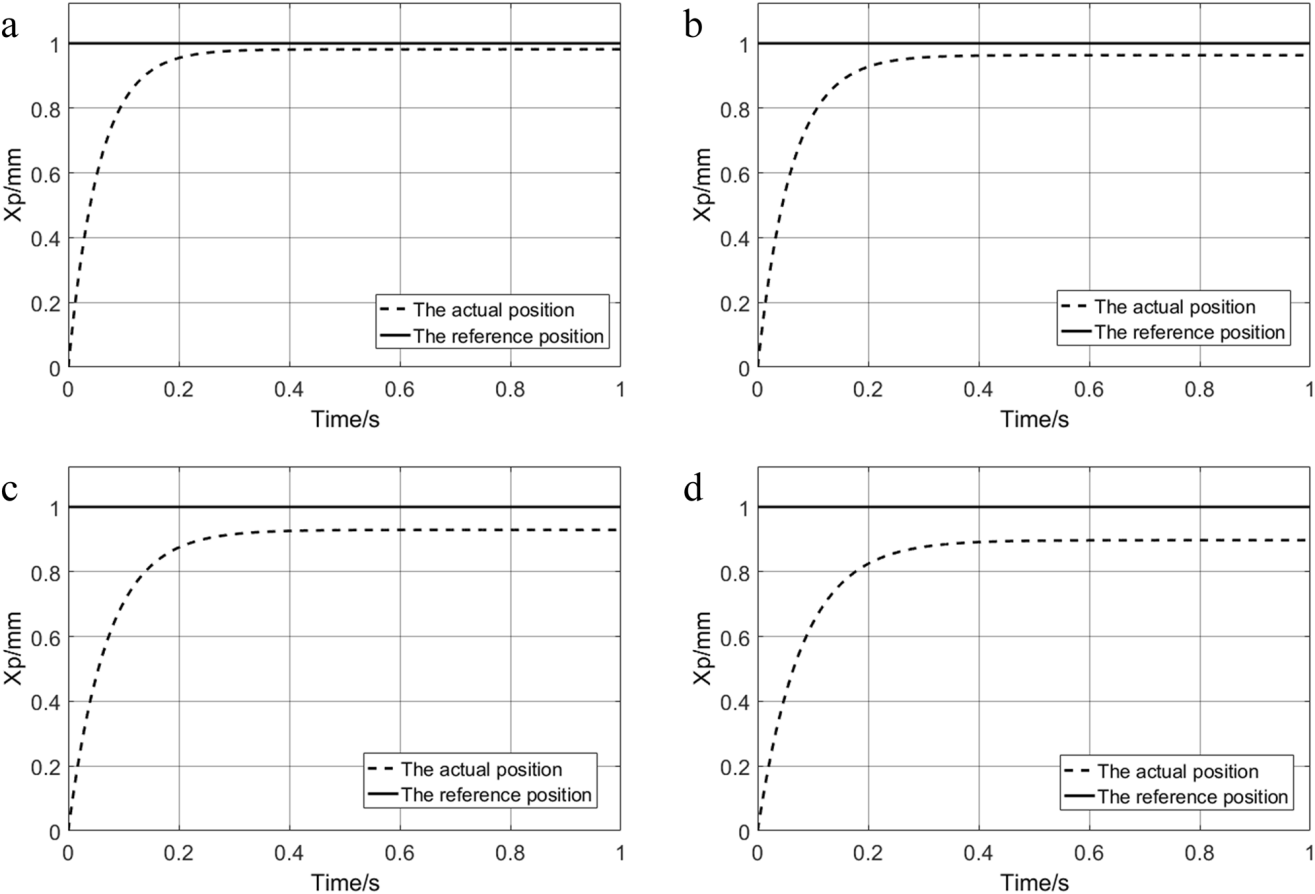
The response curve of different load stiffness: (**a**) 1.5 × 10^9^ N/m; (**b**) 3 × 10^9^ N/m; (**c**) 6 × 10^9^ N/m; (**d**) 9 × 10^9^ N/m.

The load stiffness of the input step signal was set as 1.5 × 10^9^ N/m, 3 × 10^9^ N/m, 6 × 10^9^ N/m and 9 × 10^9^ N/m respectively for simulation experiments, and different system response curves are obtained as shown in Fig. 5. With the increase in load stiffness, the time for the system to reach a steady state becomes longer, and the steady-state error also increases. When the load stiffness is 9 × 10^9^ N/m, the time for the system to reach a steady state is 0.55 s, and the steady-state error is 10.3%.

**Figure 6:**
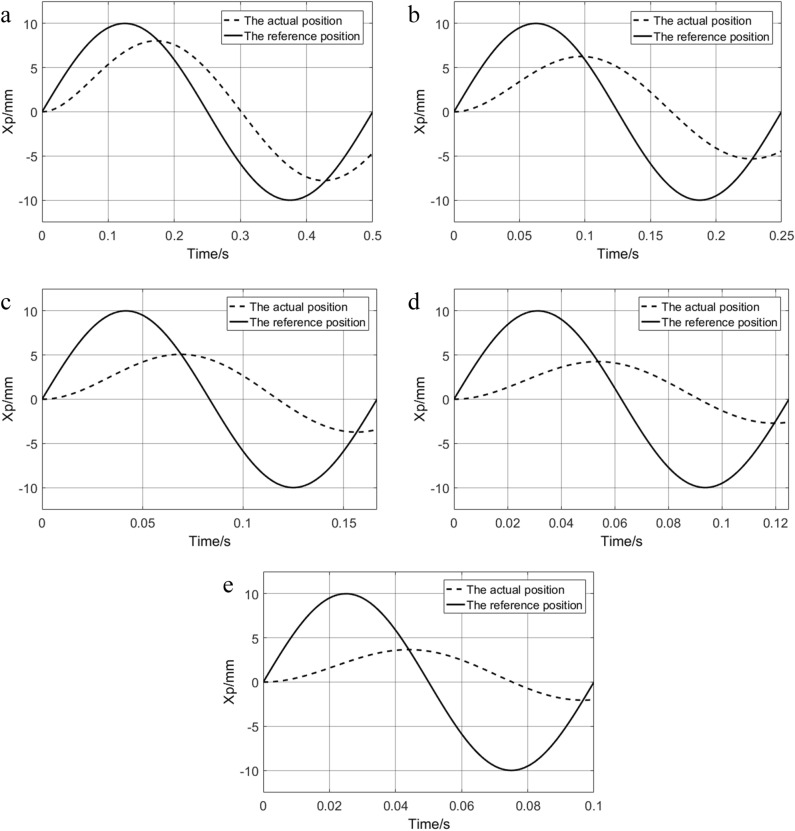
Response curve of different frequencies: (**a**) 2 HZ; (**b**) 4 HZ; (**c**) 6 HZ; (**d**) 8 HZ; (**e**) 10 HZ.

The sinusoidal signals with input frequencies of 2 HZ, 4 HZ, 6 HZ, 8 HZ, and 10 HZ are simulated, and the results are shown in Fig. 6. With the increase of input frequency, the phase lag and amplitude attenuation of the system increase continuously.

Through the above analysis, it is found that the system has the following problems to be solved. (1) poor steady-state accuracy and long adjustment time. (2) weak ability to resist external interference. (3) Under the condition of large load stiffness, the steady-state error is large and the response speed is slow. (4) The phase lag and amplitude attenuation are obvious at high frequency.

## Fuzzy control RBF neural network PID controller design

PID control algorithm is a very mature control strategy, with the advantages of simplicity and high reliability, so in the field of industrial control, PID control is the most mature and widely used control means at present. With the development of industry, the controlled object becomes more and more complex. The parameters of the control object have the characteristics of time-varying and nonlinear, etc. The PID parameters need to be selected by trial and error or relying on experience, and when the parameters of the controlled object change, the PID parameters need to be re-selected, so it is difficult for PID control to achieve the expected effect. Therefore, because of the above limitations of PID control, the combination of radial basis function (RBF) neural network and PID controller can realize the online regulation of PID parameters and improve the performance of the control system^33^.

**Figure 7:**
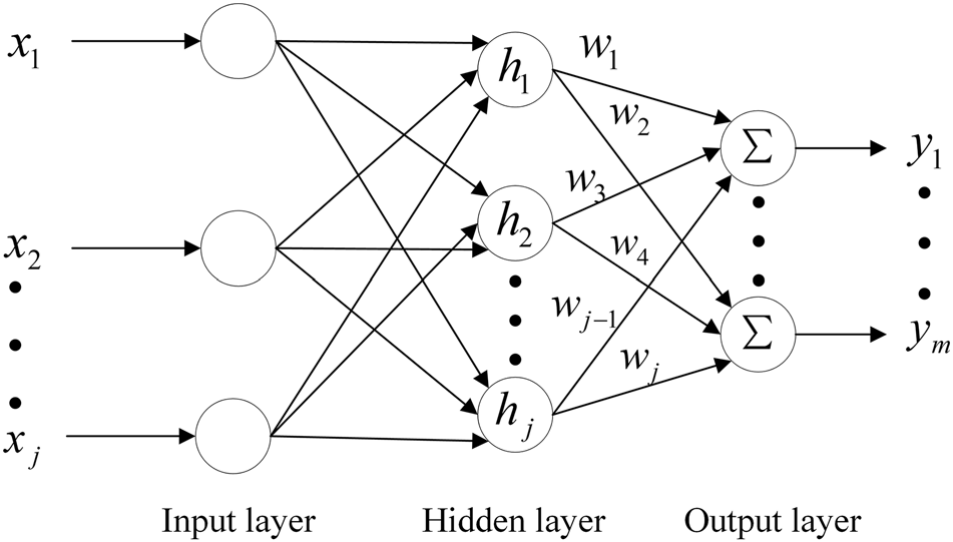
Structure diagram of RBF neural network.

RBF neural network is mainly composed of three layers, input layer, implicit layer, and output layer, and its specific network structure is shown in Fig. 7.

Input layer: input three neuron nodes, the input parameter is $$x = \left[ {\begin{array}{*{20}c} {u(t - 1)} & {y(t)} & {y(t - 1)} \\ \end{array} } \right]^{T}$$, $y(t)$ is the output of the system.

Hidden layer: $c_{j}$ is the center vector of hidden layer nodes, $b_{j}$ is the basis width vector of hidden layer nodes, and $h_{j}$ is the radial basis vector. The Gaussian basis function of the hidden layer is as follows:

13 $$ h_{j} = \exp \left[ { - \frac{{\left\| {x - c_{j} } \right\|^{2} }}{{2b_{j}^{2} }}} \right] $$

Output layer: $w_{j}$ is the weight from the hidden layer unit to the output node, $y_{m} (t)$ is the RBF network identification output, and the expression of the weighted sum of hidden layer node output as follows:

14 $$ y_{m} = h_{1} w_{1} + h_{2} w_{2} + \cdots h_{j} w_{j} $$

The performance index of neural networks is $\frac{1}{2}(y(t) - y_{m} (t))^{2}$.

Update the output weight $w_{j}$, node center $c_{j}$, and base width vector ${b}_{j}$ with gradient descent:

15 $$ w_{j} (t) = w_{j} (t - 1) + \alpha (y(t) - y_{m} (t))h_{j} + \beta (w_{j} (t - 1) - w_{j} (t - 2)) $$

16 $$ c_{ji} (t) = c_{ji} (t - 1) + \alpha (y(t) - y_{m} (t))w_{j} \frac{{x_{i} - c_{ji} }}{{b_{j}^{2} }} + \beta (c_{ji} (t - 1) - c_{ji} (t - 2)) $$

17 $$ b_{j} (t) = b_{j} (t - 1) + \alpha (y(t) - y_{m} (t))w_{j} h_{j} \frac{{\left\| {x - c_{j} } \right\|^{2} }}{{b_{j}^{3} }} + \beta (b_{j} (t - 1) - b_{j} (t - 2)) $$

where $\alpha$ is the learning rate, $\beta$ is the momentum factor. PID parameter adjustment algorithm:

18 $$ e(t) = r(t) - y(t) $$

The inputs for the PID controller are as follows:

19 $$ X_{c} (1) = e(t) - e(t - 1) $$

20 $$ X_{c} (2) = e(t) $$

21 $$ X_{c} (3) = e(t) - 2e(t - 1) + e(t - 2) $$

Adjust PID parameters by gradient descent method:

22 $$ \Delta k_{p} = \alpha_{P} e(t)\frac{\partial y(t)}{{\partial u}}X_{c} (1) $$

23 $$ \Delta k_{i} = \alpha_{I} e(t)\frac{\partial y(t)}{{\partial u}}X_{c} (2) $$

24 $$ \Delta k_{d} = \alpha_{D} e(t)\frac{\partial y(t)}{{\partial u}}X_{c}^{{}} (3) $$

where $\alpha_{P}$, $\alpha_{I}$ and $\alpha_{D}$ are PID parameter learning rate, $\Delta k_{p}$, $\Delta k_{i}$ and $\Delta k_{d}$ are PID parameter adjustment amount, $\frac{\partial y(t)}{{\partial u}}$ is Jacobin information.

25 $$ \frac{\partial y(t)}{{\partial u}} = \sum\limits_{j = 1}^{m} {w_{j} h_{j} \frac{{c_{j} - u(t - 1)}}{{2b_{j}^{2} }}} $$

The output of the incremental PID control algorithm is as follows:

26 $$ u(t) = u(t - 1) + (k_{p} + \Delta k_{p} )X_{c} (1) + (k_{i} + \Delta k_{i} )X_{c} (2) + (k_{d} + \Delta k_{d} )X_{c} (3) $$

**Figure 8:**
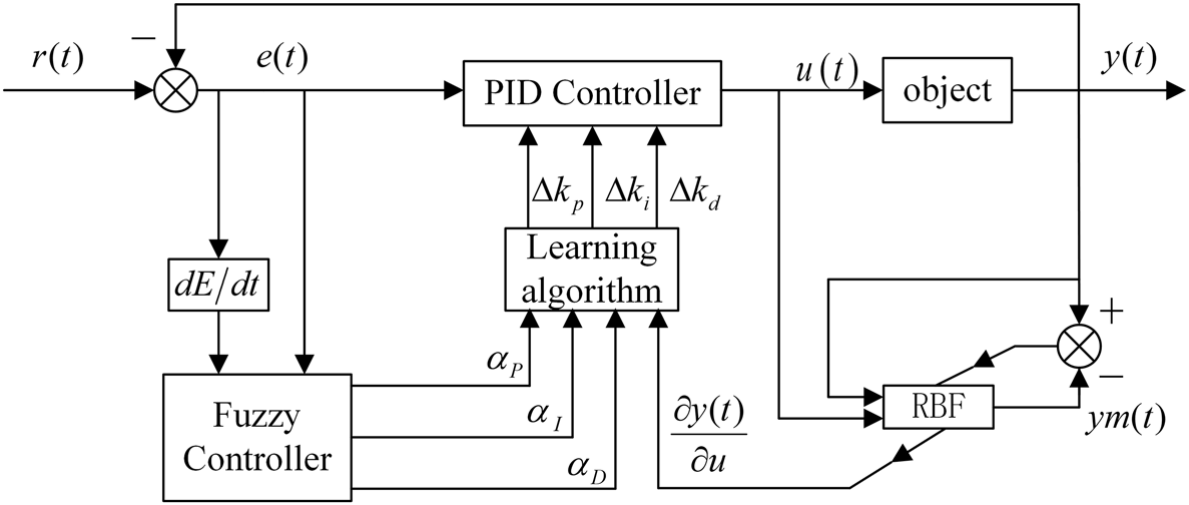
FUZZY-RBF-PID controller structure diagram.

The RBF neural network can adjust PID parameters online, but the initial PID learning rate parameters set by the RBF neural network cannot be changed, and the parameters are difficult to select. This paper uses fuzzy rules to adjust the parameters online. Fuzzy rules are used to adjust the PID parameter learning rate ($\alpha_{P}$, $\alpha_{I}$, $\alpha_{D}$). Detect deviation $e$ and deviation change rate *de*de during operation. The parameters $\alpha_{P}$, $\alpha_{I}$ and $\alpha_{D}$ were adjusted online in real time according to fuzzy rules and membership functions, and the obtained parameters $\alpha_{P}$, $\alpha_{I}$ and $\alpha_{D}$ were input into the RBF neural network learning algorithm to complete the adjustment of PID parameters. The structure of the RBF-PID controller under fuzzy rules is shown in Fig. 8.

Fuzzy logic is to build fuzzy sets so that an input may belong to several sets. The proportion of the input in different sets is different so that the input value is fuzzified. Then the set is interpreted by the fuzzy rule knowledge base to obtain a certain output value, and the defuzzification process is completed^34^.

**Figure 9:**
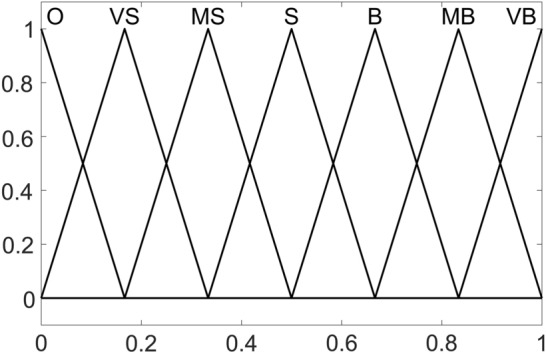
Membership function of *α*_*P*_.

The control deviation and deviation change rate of the controller are used as the input of the fuzzy controller, and the PID parameter learning rate $\alpha_{P}$, $\alpha_{I}$ and $\alpha_{D}$ are taken as the output of the controller to adjust the PID parameter learning rate. When the deviation and the change rate of the deviation are large, the learning rate of the PID parameters should be large; when the deviation and the change rate of the deviation are small, the learning rate of the PID parameters should be small. When the learning rate of PID parameters is large, the PID parameters change faster, that is, the degree of change of PID parameters is large, that is to say when the deviation changes greatly, a larger learning rate of PID parameter is required. Therefore, for the adjustment of the learning rate of PID parameters, the sensitivity of the deviation rate $de$ is higher than that of the deviation $e$. The fuzzy set of deviation is {PB(positive big), ZO(zero), and NB(negative big)}. The fuzzy set of the deviation change rate is {PB(positive big), PM(positive middle), PS(positive small), ZO(zero), NS(negative small), NM(negative middle), and NB(negative big)}. The fuzzy set of PID parameter learning rate ($\alpha_{P}$, $\alpha_{I}$ and $\alpha_{D}$) is {O(zero), VS(very small), MS(middle small), S(small), B(big), MB(middle big), VB(very big)}, and its membership function is shown in Fig. 9. Since there is no negative value for the PID parameter learning rate, the discrete domain is [0,1] and the actual domain is [0,60], [0,14.5], and [0,20], respectively.

After completing the process of fuzzification of input and defuzzification of output, it is necessary to establish a fuzzy rule of fuzzy logic. The quality of fuzzy rules often affects the results of fuzzy reasoning and the accuracy of controller^35^. The fuzzy rule table is shown in Table 2 as follow.

**Table 2:** Fuzzy rule table.

*e*	*de*						
	NB	NM	NS	ZO	PS	PM	PB
NB	MB	B	MS	VS	MB	VB	VB
ZO	S	MS	VS	O	VS	MS	S
PB	VB	VB	MB	VS	MS	B	MB

Formulation of fuzzy rules: When the rate of deviation change is large, a large learning rate of the PID parameter is required. Specifically, when the deviation rate of change is negative big, the deviation is positive big or the deviation rate of change is positive big, the deviation is negative big, the signal deviates from the desired position and is in the process of retracement, so a large learning rate of PID parameter is required to complete the retracement process quickly. When the deviation rate of change is negative big, the deviation is negative big, or the deviation rate of change is positive big, the deviation is positive big, the signal deviates from the desired position and continues to deviate, and a large PID parameter learning rate is required to quickly adjust the PID so that it rises slowly and quickly retraces. Conversely, when the deviation from the desired position is small, a small PID parameter learning rate is required to prevent overshoot and oscillation. The deviation and the deviation change rate are input into the fuzzy controller to fuzzify it. After setting fuzzy rules and the defuzzification process, the learning rate of PID parameters is obtained. Combined with the Jacobin information output by the RBF neural network, three different PID parameter adjustment amounts $\Delta k_{p}$, $\Delta k_{i}$ and $\Delta k_{d}$ are obtained by the learning algorithm, and then the PID parameters can be adjusted online. The controller can eliminate the steady-state error, reduce the adjustment time, and improve the control accuracy.

Rewrite the system transfer function (5) as:

27 $$ \frac{Y}{U} = \frac{{a_{1} }}{{s^{3} + a_{2} s^{2} + a_{3} s + a_{4} }} $$

where $Y$ is the system output, $U$ is the control quantity, and $a_{i} (i = 1,2,3,4)$ are the system parameters. The following system can be obtained:

28 $$ \left\{ {\begin{array}{*{20}l} {\dddot x = - a_{2} \ddot{x} - a_{3} \dot{x} - a_{4} x + a_{1} U} \hfill \\ {Y = x} \hfill \\ \end{array} } \right. $$

where, $x$ is the system state, $U = k_{1} e + k_{2} \int_{0}^{t} e d\tau + k_{3} \dot{e}$ is the control quantity, $k_{1}$, $k_{2}$, and $k_{3}$ are PID controller parameters and the system error is defined as:

29 $$ e = x_{d} - x $$

where $e$ is the system error and $x_{d}$ is the desired signal. From (3) :

30 $$ \begin{gathered} \frac{{d^{3} (x_{d} - x)}}{dt} = - a_{2} \ddot{x} - a_{3} \dot{x} - a_{4} x + a_{1} U \\ - \dddot x = a_{2} \ddot{x} + a_{3} \dot{x} + a_{4} x - a_{1} U \\ \end{gathered} $$

Then:

31 $$ \dddot e = - a_{2} \ddot{e} - (a_{3} + a_{1} k_{3} )\dot{e} - (a_{4} + a_{1} k_{1} )e - a_{1} k_{2} \int_{0}^{t} e d\tau + a_{4} x_{d} $$

Differentiating both sides of Eq. (6) has:

32 $$ \ddddot e + a_{2} \dddot e + (a_{3} + a_{1} k_{3} )\ddot{e} + (a_{4} + a_{1} k_{1} )\dot{e} + a_{1} k_{2} e = 0 $$

According to Routh–Hurwitz stability criterion:

33 $$ \begin{gathered} \, \ddddot e + a_{2} \dddot e + (a_{3} + a_{1} k_{3} )\ddot{e} + (a_{4} + a_{1} k_{1} )\dot{e} + a_{1} k_{2} e = 0 \hfill \\ s^{4} \, 1 \, (a_{3} + a_{1} k_{3} ) \, a_{1} k_{2} \hfill \\ s^{3} \, a_{2} \, (a_{4} + a_{1} k_{1} ) \, 0 \hfill \\ s^{2} \, b_{1} \, b_{2} \, 0 \hfill \\ s^{1} \, c_{1} \, 0 \hfill \\ s^{0} \, d_{1} \hfill \\ \end{gathered} $$

where, $b_{1} = \frac{{a_{2} (a_{1} k_{3} + a_{3} ) - (a_{4} + a_{1} k_{1} )}}{{a_{2} }}$, $b_{2} = a_{1} k_{2}$, $c_{1} = \frac{{b_{1} (a_{4} + a_{1} k_{1} ) - a_{2} b_{2} }}{{b_{1} }}$, $d_{1} = b_{2}$. Only $b_{1} > 0$, $c_{1} > 0$, and $d_{1} > 0$ need to be satisfied to ensure the stability of the error system. Therefore, the above goal can be achieved by choosing the appropriate $k_{1}$, $k_{2}$, and $k_{3}$. When the system parameters change or are disturbed by the load force, the PID controller parameters need to be corrected. In this paper, the fuzzy rules and the RBF neural network are used to complete this adjustment process, which can ensure that PID controller parameters meet the requirements at all times. Therefore, the proposed method can ensure the stability of the system and the convergence of the error.

## System simulation results and analysis

**Figure 10:**
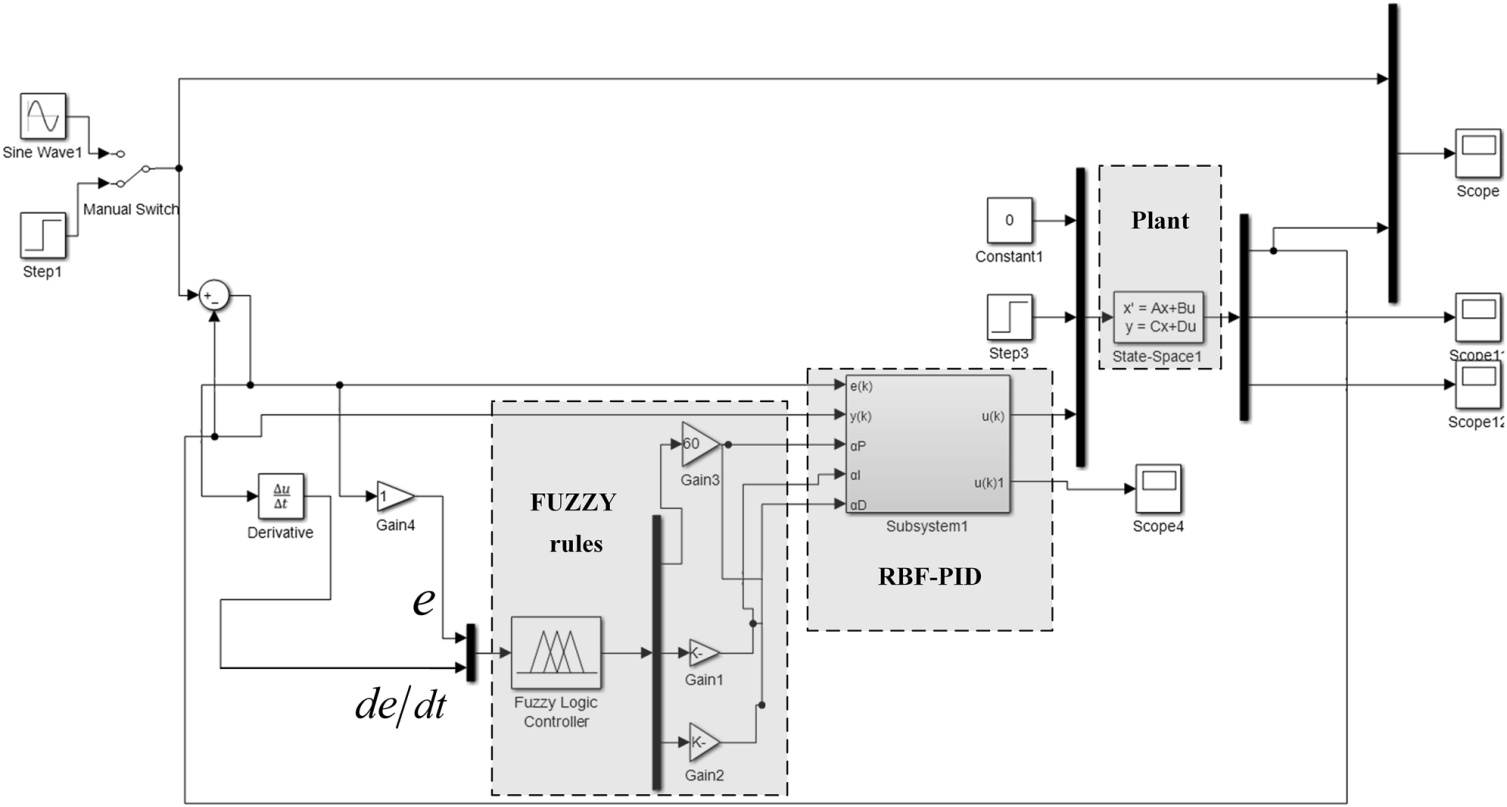
Simulink control diagram.

The Simulink control diagram is shown in Fig. 10.

The simulation system is built in Matlab/Simulink software, and step signal and sinusoidal signal are applied to the system to verify the feasibility of the designed control strategy.

**Figure 11:**
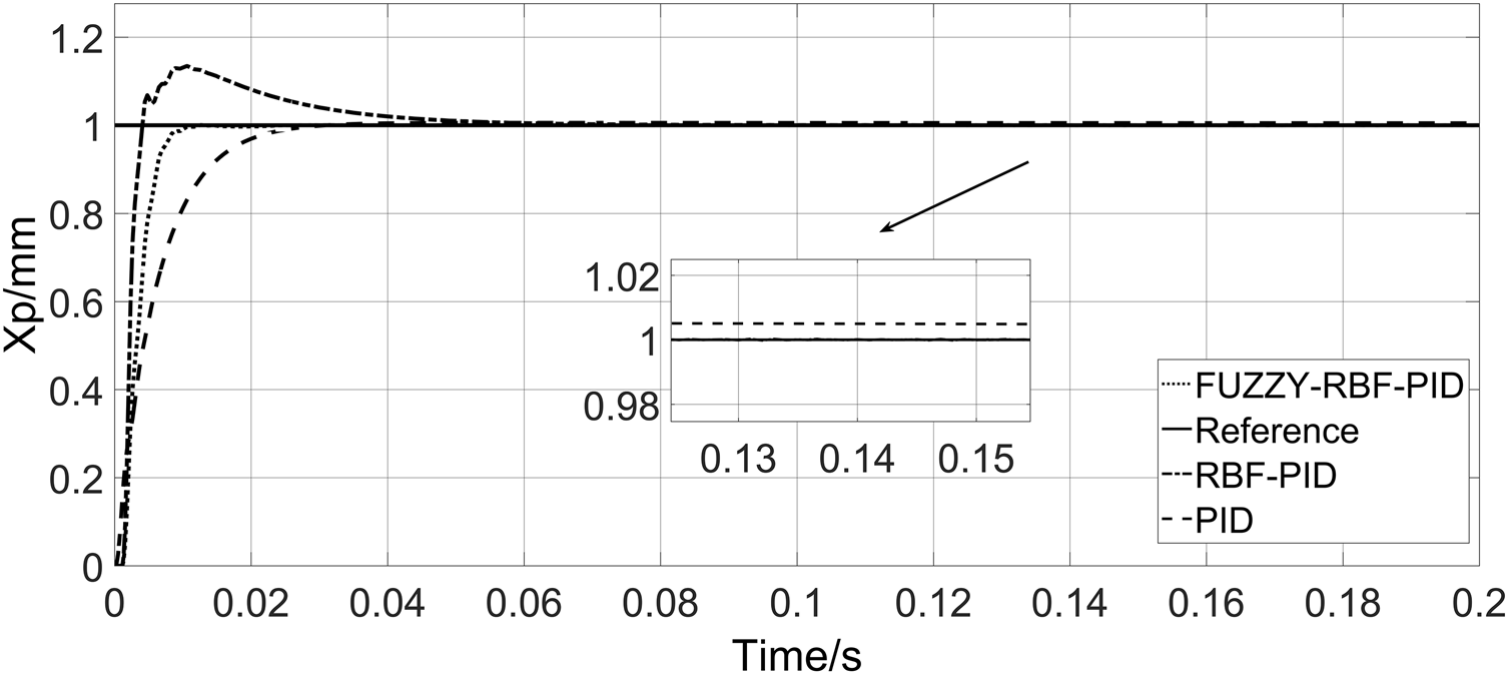
System no-load response curve.

Three kinds of controllers, traditional PID controller (PID), traditional RBF neural network PID controller (RBF-PID), and radial basis function neural network PID controller under FUZZY rules (FUZZY-RBF-PID), are used to simulate the system under the conditions of no-load, load and large load stiffness. The steady-state accuracy, regulation time, and anti-interference ability of different control strategies are compared. Under the no-load condition, the response curve of the system is shown in Fig. 11.

Observation of Fig. 11 shows that the steady-state time of PID is 0.591 s, without overshoot, but with a certain steady-state error. The steady-state time of RBF-PID is 0.865 s, and the overshoot is 13.4%. The steady-state accuracy of the control is higher than that of PID, but the overshoot is larger, and the time to reach the steady state is longer. The steady-state time of FUZZY-RBF-PID is 0.0131 s, without overshoot, and the steady-state error can be effectively eliminated. The table of system no-load response characteristic values is shown in Table 3.

**Table 3:** System no-load response characteristic value table.

Controller	Steady-state time (s)	Overshoot (%)
PID	0.591	0
RBF-PID	0.865	13.4
FUZZY-RBF-PID	0.0131	0

**Figure 12:**
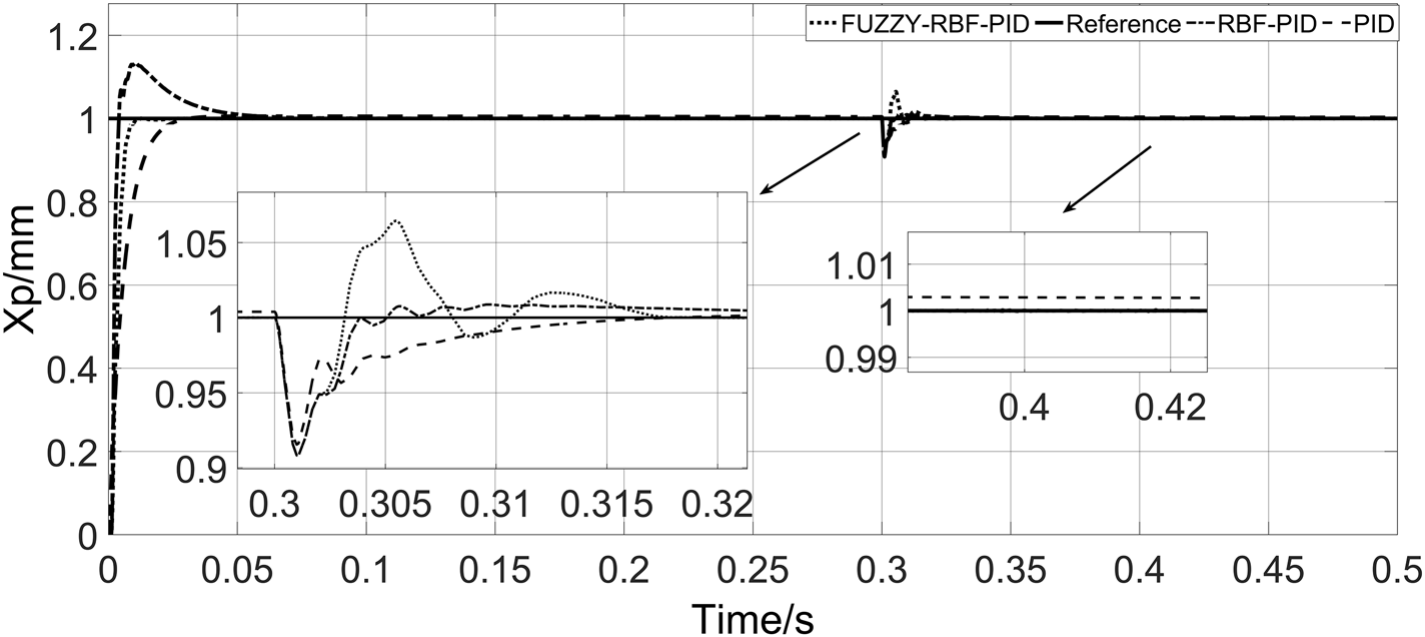
System load response curve.

To verify the anti-interference ability of the system, an external load is applied at 0.3 s, and the response curve of the system is shown in Fig. 12.

As shown in Fig. 12, after the external load is input, the adjustment time of PID is 0.0375 s, the amplitude fluctuation is 8.982%, and there is still a certain steady-state error. The amplitude fluctuation of RBF-PID is 9.174%, and the steady-state accuracy is higher, but the adjustment time is longer, 0.625 s. The amplitude fluctuation of FUZZY-RBF-PID is slightly larger, 9.177%, but the steady-state error can be effectively eliminated, and the adjustment time is shorter, 0.0195 s. According to the above results, compared with the other two control strategies, the FUZZY-RBF-PID controller eliminates steady-state errors and allows faster adjustments when exposed to disturbed loads. The system load response characteristic values table is shown in Table 4.

**Table 4:** System load response characteristic value table.

Controller	Load adjustment time (s)	Amplitude fluctuation (%)
PID	0.0375	8.982
RBF-PID	0.0625	9.174
FUZZY-RBF-PID	0.0195	9.177

**Figure 13:**
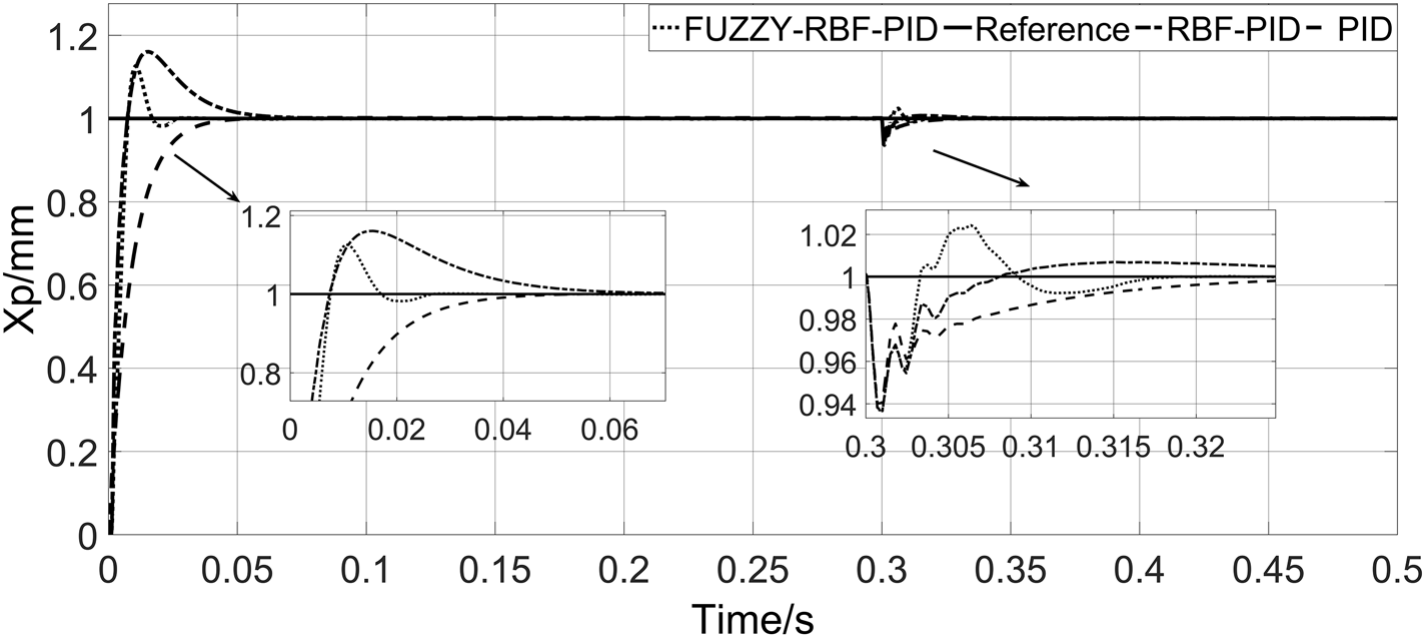
Response curve with load stiffness of 9 × 10^10^ N/m.

The response curve of the system with load stiffness 9 × 10^10^ N/m and load disturbance is shown in Fig. 13.

As shown in Fig. 13, under the condition of load stiffness, the time for PID to reach the steady state is 0.0804 s, without overshoot, and there is a certain steady-state error. RBF-PID has an overshoot of 16%, with higher steady-state accuracy but a longer steady-state time of 0.096 s. FUZZY-RBF-PID has an overshoot of 12.6%, which can effectively eliminate steady-state errors and has a shorter adjustment time of 0.0394 s. When disturbed, the adjustment time of PID is 0.072 s, there is still some steady-state error, and the adjustment time of RBF-PID is 0.0744 s. The adjustment time for FUZZY-RBF-PID is 0.019 s. Under the condition of large load stiffness, FUZZY-RBF-PID has a certain amount of overshoot, but the steady-state time and regulation time are short, and the steady-state error can be eliminated. The system response characteristic value table is shown in Table 5.

**Table 5:** Characteristic values of system response.

Controller	Steady-state time (s)	Overshoot (%)	Load adjustment time (s)	Amplitude fluctuation (%)
PID	0.0804	0	0.0072	6.12
RBF-PID	0.096	16	0.0744	6.34
FUZZY-RBF-PID	0.0394	12.6	0.019	6.39

The system has phase lag and amplitude attenuation under high-frequency signals and has a large tracking error. The sinusoidal signals with frequencies of 1HZ and 10HZ were selected to verify the ability of the designed control strategy to solve the above problems.

**Figure 14:**
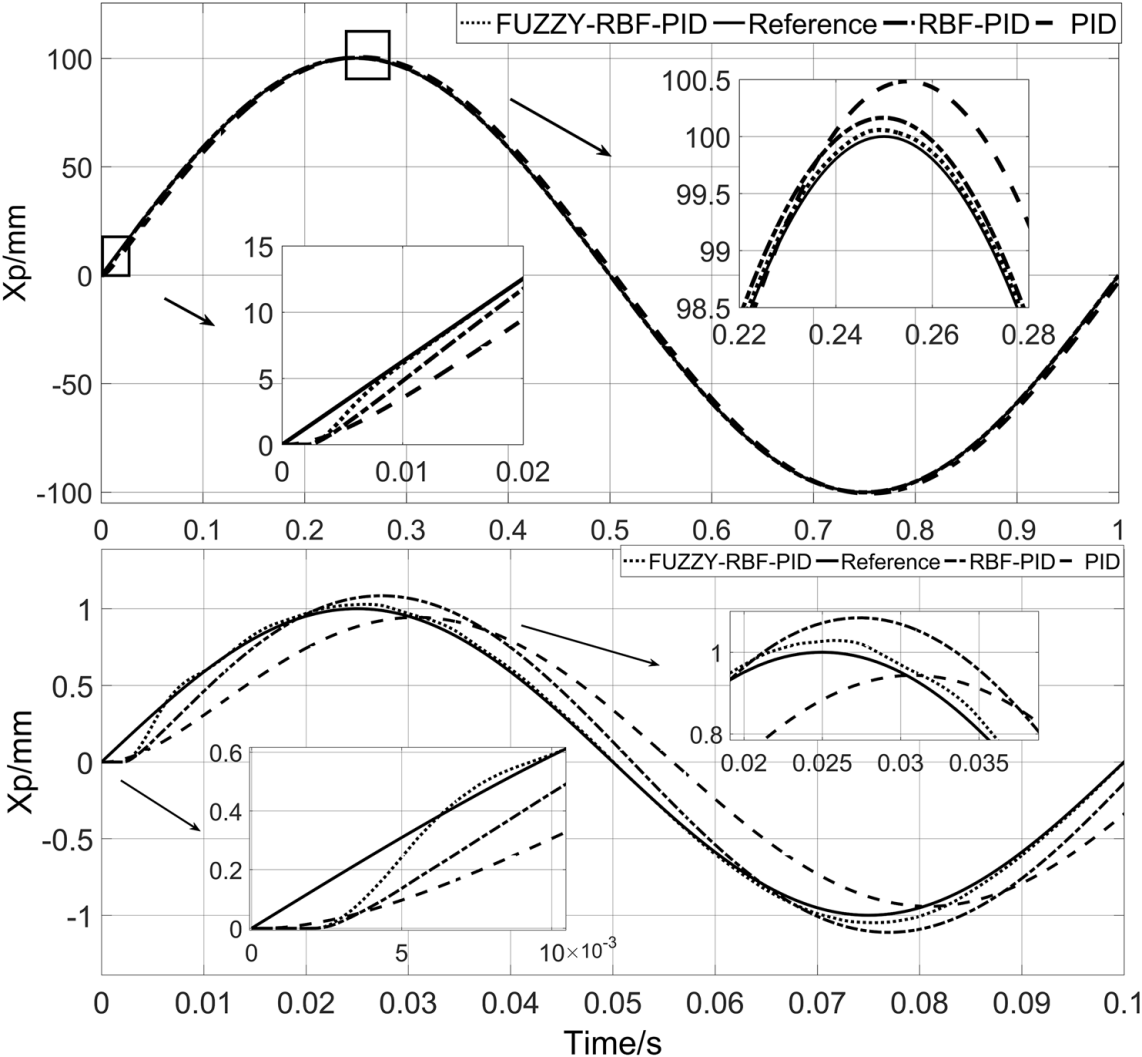
system response curve with 1HZ and 10HZ.

The response curve of the system is shown in Fig. 14. The characteristic values of the sinusoidal response with 10HZ of the system are shown in Table 6.

**Table 6:** Characteristic values of system sinusoidal response.

Controller	Tracking error (mm)
PID	− 0.36–0.36
RBF-PID	− 0.16–0.172
FUZZY-RBF-PID	− 0.034–0.056

When the frequency is changed from 1 to 10 Hz, the phase lag of the PID controller becomes more obvious. At 1 Hz, neither RBF-PID nor the proposed method has a phase lag phenomenon, which is due to the slow change of the desired signal, and both of them can adjust the PID parameters near the optimal value, and at 10 Hz, RBF-PID has phase lag phenomenon, while the proposed method still has good tracking effect. This is because at 10 HZ, the change speed of the desired signal becomes faster, and the RBF-PID adjusts the PID parameters with a fixed learning rate, which cannot keep up with the change speed of the desired signal, whereas the proposed method can modify the learning rate according to the change speed of the desired signal, and then adjust the change speed of the PID parameters. This also proves the effectiveness of the proposed method. According to the above results and Table 6, it can be concluded that compared with the other two control strategies, the designed FUZZY-RBF-PID control strategy has good performance, higher tracking accuracy, faster adjustment speed, and better anti-interference ability.

## Conclusion

Aiming at the electro-hydraulic position servo system with uncertain parameters, a mathematical model is established and its characteristics are analyzed. Based on this, a radial basis function neural network PID controller under fuzzy rules is designed. Compared with the PID controller and RBF-PID controller, simulation analysis was carried out. Under the no-load condition, the steady-state time of the system under FUZZY-RBF-PID control is 0.0131 s, without overshoot, and the steady-state error can be effectively eliminated. Compared with the other two controllers, FUZZY-RBF-PID can recover the steady-state value faster under load state and large load stiffness state. Under the sinusoidal signal with an input frequency of 10HZ, FUZZY-RBF-PID has no obvious phase lag phenomenon, and the tracking error is smaller than the other two controllers. The control strategy designed in this paper can improve the performance of the electro-hydraulic position servo system to a greater extent.

## Data Availability

All data generated or analysed during this study are included in this published article.
